# Effect of intentional weight loss through dietary intervention on cognition: The MIND Trial Study

**DOI:** 10.1002/alz.087624

**Published:** 2025-01-09

**Authors:** Klodian Dhana, Jennifer Ventrelle, Vincent J Carey, Neelum T. Aggarwal, Konstantions Arfanakis, Frank Sacks, Lisa L. Barnes

**Affiliations:** ^1^ Rush University Medical Center, Chicago, IL USA; ^2^ Rush Institute for Healthy Aging, Chicago, IL USA; ^3^ Harvard Medical School, Boston, MA, Boston, MA USA; ^4^ Rush Alzheimer’s Disease Center, Chicago, IL USA; ^5^ Illinois Institute of Technology, Chicago, IL USA; ^6^ Harvard T. H. Chan School of Public Health, Boston, MA USA; ^7^ Rush Alzheimer’s Disease Center, Rush University Medical Center, Chicago, IL USA

## Abstract

**Background:**

With the increasing prevalence of obesity and its negative consequences on health, weight management has emerged as a priority for public health. We conducted a study to investigate the association of intentional weight loss through dietary intervention on cognition among older adults participating in the MIND trial.

**Method:**

MIND trial enrolled overweight older adults aged 65‐85 years with a family history of dementia but without cognitive impairment and self‐reporting a suboptimal diet. Participants were randomized to the MIND diet or a control (usual) diet for 3 years; both diets promoted weight loss through mild caloric restriction. Of 604 individuals enrolled in the MIND trial, 520 had measured weight at the baseline and year 3 visit and were included in the analysis. We computed the percentage of weight loss based on baseline and last assessment and grouped individuals into 4 categories: weight gainers, <5% weight loss, 5‐10% weight loss, and >10% weight loss. Linear mixed‐effect models were used to estimate the 3‐year change in global cognitive scores. Models were adjusted by age, sex, race, apolipoprotein E (APOE) e4 allele, baseline BMI, and dietary assignment.

**Result:**

The mean age was 70 years, 65% were women, and the mean BMI was 34 kg/m^2^. During 3 years of dietary intervention, 78.5% of participants lost weight. The average weight loss ranged from 2.3 kg to 15kg. Compared to people who gained weight, those with >10% weight loss significantly improved their cognitive scores by 0.103 SD/units (95%CI 0.008, 0.199) at year 3 visit. In addition, individuals who lost weight had improved plasma levels of inflammation, lipids, and glucose during the trial period. Among people in the MIND diet group, those with >10% weight loss significantly improved their cognitive scores by 0.171 SD/units (95%CI 0.028, 0.314) compared to people who gained weight; there was no significant association in the control diet group.

**Conclusion:**

Among overweight and cognitively unimpaired individuals at the baseline with a family history of dementia, a weight loss of 10% (average 15 kg) or more during 3 years of dietary interventions was associated with improved cognitive scores when compared to people who gained weight.

